# Evaluating the Quality and Safety of Ambient Digital Scribe Platforms Using Simulated Ambulatory Encounters

**DOI:** 10.1016/j.mcpdig.2025.100292

**Published:** 2025-10-09

**Authors:** Taylor N. Anderson, Vishnu Mohan, David A. Dorr, Raj M. Ratwani, Joshua M. Biro, Jeffrey A. Gold

**Affiliations:** a Division of Informatics, Clinical Epidemiology, and Translational Data Science, Oregon Health & Science University, Portland, OR; b Department of Internal Medicine and Geriatrics, Oregon Health & Science University, Portland, OR; c Division of Pulmonary and Critical Care Medicine, Oregon Health & Science University, Portland, OR; d Medstar Health National Center for Human Factors in Healthcare, Washington, DC

## Abstract

**Objective:**

To evaluate and compare the quality and safety of ambient digital scribe (ADS) platforms using simulated ambulatory encounters.

**Methods:**

Five ADS platforms were evaluated using audio recordings of fourteen simulated clinical encounters. Audio recordings were played on a laptop computer and captured by ADS platforms on a mobile phone. Generated transcripts were compared to professional transcriptions. Clinical notes were graded using rubrics of key elements for each case. Note errors were classified as omission, commission, or partially correct. Potential clinical harm was assessed using the agency for healthcare research and quality harm scale. Note quality was assessed using the 9-item Physician Documentation Quality Instrument (range 9-45). Statistical comparisons included Friedman and χ^2^ tests with a correction for multiple comparisons.

**Results:**

Transcripts generated by platforms A through D contained an average of 13.9 (95% CI, 6.0-17.5) errors, with 19.5% of the transcript errors transmitted to the clinical note (95% CI, 6.6%-28.8%). For clinical notes, mean percent error across platforms was 26.3% (95% CI, 17.0%-31.0%) with a significantly higher proportion of errors in notes generated by platform E (*P*<.0053 for all comparisons). Of correctly reported elements, only 35.8%±11.3% were consistently correct across all platforms. An average of 3.0 (95% CI, 0-4, range 0-21) errors per case had potential for moderate-to-severe harm. The mean physician documentation quality instrument–9 score was 36±4, with significant variation between platforms.

**Conclusion:**

Clinical notes generated by ADS platforms using simulated encounters reports important inter-platform and intra-platform variability in accuracy and quality. These findings indicate a need for standardized, objective evaluation and reporting.

Documentation burden remains a considerable challenge among medical providers and is a key area of focus in the effort to decrease clinician burnout. In one study, family medicine physicians spent nearly 6 hours per day in the electronic health record, with 25% of this time allocated to documentation.^1^ Across ambulatory specialties, physicians report a daily average of nearly 2 hours of documentation time outside of normal work hours, with around 10% reporting over 4 hours per day.^2^ These observations indicate a widespread lack of sufficient documentation time in the clinical workday, which has been associated with increased odds of provider burnout.^3^

In recent years, ambient digital scribe (ADS) platforms have shown promise for alleviating this growing burden of clinical documentation. These platforms use ambient speech recognition (ASR) to transcribe clinical encounters in real time, subsequently generating structured notes through large language model (LLM) processing. Recent studies have suggested that ADS implementation may reduce documentation burden, enhance provider wellbeing, and improve patient satisfaction.^4^, ^5^, ^6^, ^7^, ^8^

Uptake of this technology in the clinical setting has been rapid, despite minimal regulatory oversight and a paucity of quality and safety data. As of early 2024, nearly a third of medical groups, defined as collectives of medical providers operating under the same administrative structure, reported adoption of ADS platforms.^9^ This consumer demand has been accompanied by the rollout of multiple ADS platforms, with over 90 platforms currently available for clinical use.^10^ Although vendors frequently cite internal quality control measures, the absence of publicly available performance metrics hinders meaningful assessment of the risk-benefit profile of this technology. An objective, transparent, standardized evaluation and reporting framework is urgently needed to guide informed adoption, mitigate potential harms, and retain trust.

Despite limited objective data on the accuracy of ADS-generated content, both ASR and LLM documentation tools have been shown to introduce clinically significant errors.^11^, ^12^, ^13^ Furthermore, LLMs are vulnerable to bias and hallucination, which depend predominantly on model training data and parameters.^14^ The opaque, black box nature of ADS technology further limits user ability to anticipate and mitigate these risks. Although clinician proofreading remains the *de jure* safeguard against ADS-generated errors, early simulation studies suggest this may not be an effective safeguard.^15^ The effectiveness of relying on clinician proofreading has yet to be systematically evaluated in real-world practice.

Given the increasing prevalence of ADS systems in clinical care, a reproducible, standardized framework for objective evaluation is essential. In this study, we assessed the accuracy, quality, and safety of ADS platforms in the ambulatory setting using simulated patient encounters.

## Methods

We evaluated 4 commercially available ADS platforms (A-D) and 1 free platform (E) using audio recordings from 14 simulated ambulatory patient-provider encounters previously created for a medical scribe training curriculum.^16^ Simulated cases represented common clinical scenarios in a variety of ambulatory specialties (Supplemental Table 1, [available online at https://www.mcpdigitalhealth.org/]). Each scenario contained elements of a standard patient encounter, including (but not limited to) history of present illness, past medical history, medications, physical exam with vital signs, clinical assessment, and plan. The mean duration of the simulated cases was 13.4 minutes (range 7.9-22.1 minutes). A professional human transcription service was used to generate gold standard transcriptions from the recorded encounters. Audio recordings were played on a 2022 MacBook Pro laptop and captured by each ADS application running on an iPhone 14 Pro placed ∼1 ft away in a quiet room. Each evaluation was performed separately, with one ADS platform running at a time. Simulation testing was performed from December 2024 to February 2025 using the most current version of each platform at the time of evaluation.

We first assessed the generated transcripts by comparing them with the professional transcriptions for each case. Transcript errors were defined as substitutions, deletions, or additions with potential substantive impact on note content. These errors were further categorized as transmitted or not transmitted based on whether they impacted the generated clinical note. Minor syntactic discrepancies with no semantic impact were not classified as errors.

Clinical note quality was assessed using the 9-item Physician Documentation Quality Instrument 9-item version (PDQI-9).^17^ This validated, objective note assessment tool assigns a quality score (range: 9-45) across 9 measures scored on a 5-point Likert scale: up-to-date, accurate, thorough, useful, organized, comprehensible, succinct, synthesized, and consistent.

To assess clinical note accuracy and completeness, we utilized standardized scoring rubrics of key clinical elements previously developed by 2 clinical experts based on simulated encounter recordings and clinical context.^16^ The ADS-generated notes were manually graded based on these rubrics, with errors classified as omission, commission, or partially correct based on a previously published error taxonomy for generative artificial intelligence applications.^18^ The potential clinical harm of each error was assessed by 2 qualified clinicians (T.A. and J.G.) using the agency for healthcare research and quality (AHRQ) harm scale adapted for potential risk (Supplemental Table 2, [available online at https://www.mcpdigitalhealth.org/]).^19^ Rater agreement was defined as a scoring discrepancy of ≤1 point difference for each element. To establish consistent rating standards, the raters first performed preliminary scoring for 2 of the 14 cases and discussed discrepancies until agreement was reached. For the remaining cases, all discrepancies of ≥2 points were resolved to within a 1-point discrepancy, and the average of the 2 scores was used to determine the final harm score for each error. Percent agreement, defined as the number of elements initially scored within a 1-point discrepancy divided by the total number of elements, was calculated as 98.1%. The harm risk severity score was dichotomized as none-to-low (AHRQ score <2) and moderate-to-severe (AHRQ ≥2). Weighted average AHRQ score was calculated as the summed harm score for each case divided by the total number of case elements (range 0-4, with 0 representing no errors and 4 representing a potentially fatal error for every key element). Errors were qualitatively assessed for common etiologies and content.

Statistical analyses were performed using GraphPad Prism (version 10; GraphPad Software Inc) and Stata (version BE 18.0, StataCorp). To account for within-case pairing and nonnormal data distribution, we used the Friedman test with Dunn’s correction for overall and between-group comparisons. Chi-square tests with Bonferroni correction for multiple comparisons were used to evaluate the distribution of error subtypes. A *P*<.05 was considered statistically significant prior to Bonferroni correction.

## Results

### Transcript Evaluation

**Figure 1:**
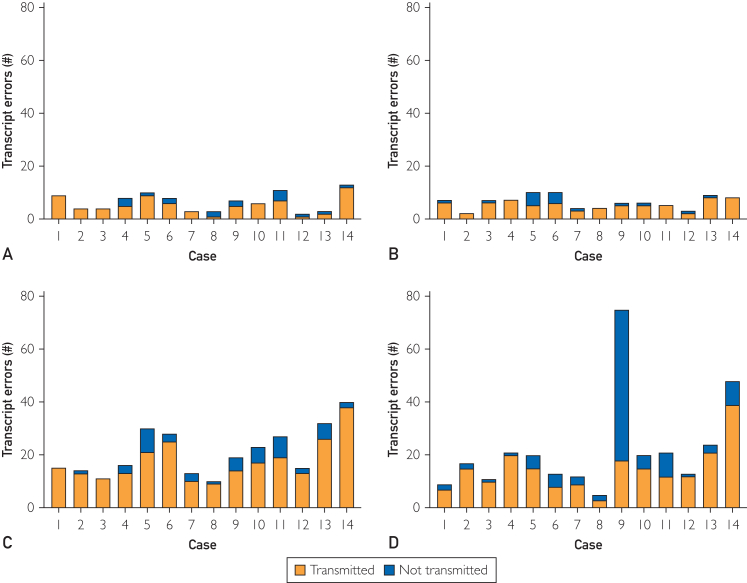
Transcript errors by case and ADS application (A-D). Errors transmitted into the clinical note are denoted in red.

We first evaluated the accuracy of generated transcripts to assess ASR performance. Platform E produced edited summaries instead of transcripts and was consequently not assessed. Transcripts generated by platforms A through D contained an average of 13.9 (95% CI, 6.0-17.5) errors per case (Table). Transcripts generated by applications C and D contained more errors than those generated by applications A and B (*P*<.0020 for all comparisons, Figure 1A-D). Across platforms, the average proportion of transcript errors transmitted to the note was 19.5% (95% CI, 6.6-28.8%), with no significant differences between platforms (Table 1).

**Table 1:** Transcript and Clinical Note Characteristics for Ambient Digital Scribe Platforms^a^^,^^b^

	Transcript		Clinical Note							
	Errors (#)	Transmitted Errors (%)	Length (Words)	Errors (%)				AHRQ ≥2 Errors (#)	Weighted AHRQ	PDQI-9
				Total	Partial	Omission	Commission			
**A**	6.5 (3.5)	21.1 (21.8)	446 (80)	16.7 (8.3)	1.6 (1.7)	13.3 (6.8)	1.9 (1.6)	2.6 (3.0)	0.16 (0.13)	35.0 (4.7)
**B**	6.3 (2.5)	15.8 (16.2)	491 (83)	23.2 (6.3)	3.5 (2.8)	17.9 (6.1)	1.9 (2.2)	1.9 (2.5)	0.17 (0.09)	38.4 (2.2)
**C**	20.9 (9.1)	15.6 (10.5)	516 (79)	22.1 (9.2)	3.9 (2.0)	17.3 (8.0)	0.9 (1.3)	1.4 (2.1)	0.14 (0.09)	39.4 (2.1)
**D**	22.1 (18.3)	25.7 (18.9)	495 (113)	23.3 (13.2)	2.8 (2.4)	18.7 (13.4)	1.8 (1.2)	3.0 (5.4)	0.20 (0.19)	35.5 (3.5)
**E**	NA	NA	271 (26)	46.1 (11.4)	5.1 (2.1)	34.1 (10.3)	6.9 (4.0)	6.2 (4.9)	0.45 (0.19)	32.3 (2.2)

a Abbreviations: AHRQ, Agency for Healthcare Research and Quality Harm Scale, score ≥2 represents potential for moderate-to-severe harm; NA, not applicable; PDQI-9, physician documentation quality instrument 9-item version.

b A-E values expressed as mean (SD).

### Clinical Note Evaluation

**Figure 2:**
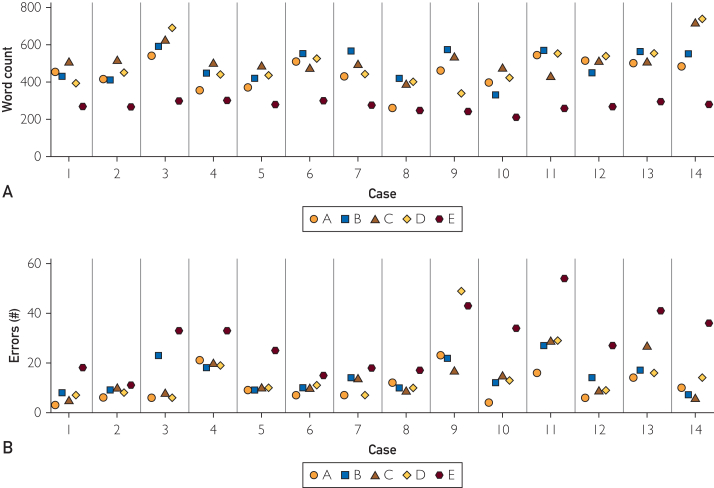
Note length (A) and errors per note (B) by case and vendor.

Evaluating clinical note length, we observed a mean length of 444±120 words across all platforms (Table 1). Notes generated by platform E were significantly shorter than those generated by platforms B, C, and D (*P*<.001), but not platform A (*P*=.060, Figure 2A). Looking at note quality, we observed a mean PDQI-9 score of 36±4 across the 5 platforms. The mean PDQI-9 score for platform C was higher than platforms A, D, and E (*P*<.028 for all comparisons); mean PDQI-9 score for platform B was also higher than platform E (*P*=.0010).

**Figure 3:**
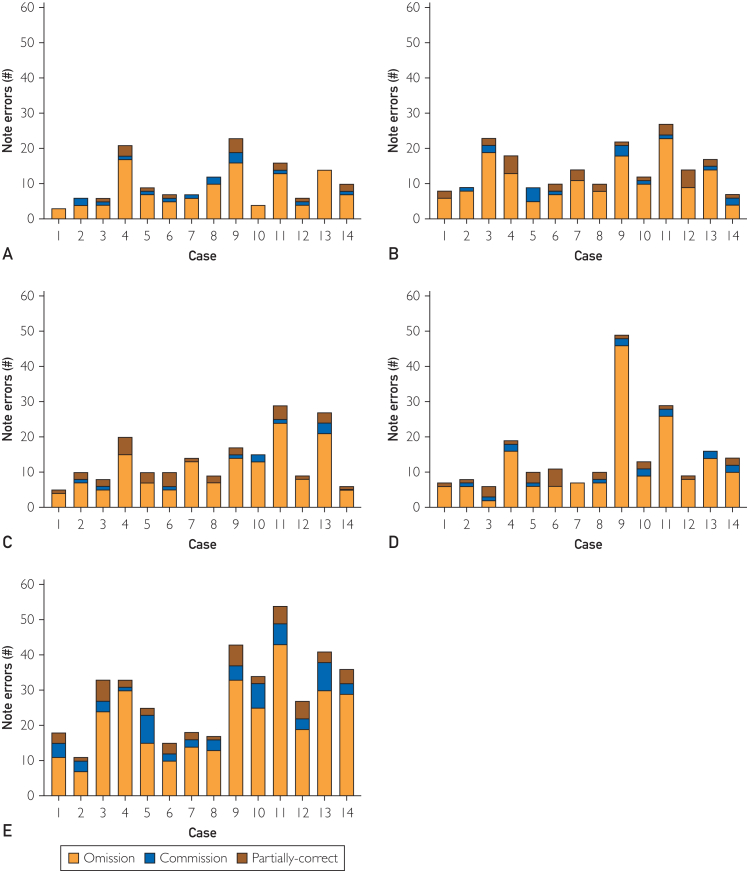
Number of errors per case according to ADS application (A-E) and error subtype (omission=white, commission=red, and partially correct=blue).

Next, we analyzed cross-platform note accuracy and errors. Across all notes for the 5 platforms, a mean of 26.3% (95% CI, 17.0%-31.0%) key clinical elements were omitted or captured erroneously. Platform E had a higher overall proportion of errors per case than platforms A-D (*P*<.0053 for all comparisons, Table 1). We observed additional variation in the number of errors between cases, with an overall average of 16.4 (95% CI, 9.0-11.0) errors per case (Figure 2B). Looking at error subtypes, errors of omission were most frequent across all platforms, comprising a mean of 76.3% (95% CI, 70.0%-83.3%) of all errors (Figure 3). The proportion of errors of omission was significantly greater than errors of commission (*P*=.022). We observed a higher proportion of errors of commission for platform E compared to platform C (*P*<.001). No significant differences in error subtype proportions were observed among the remaining platforms.

**Figure 4:**
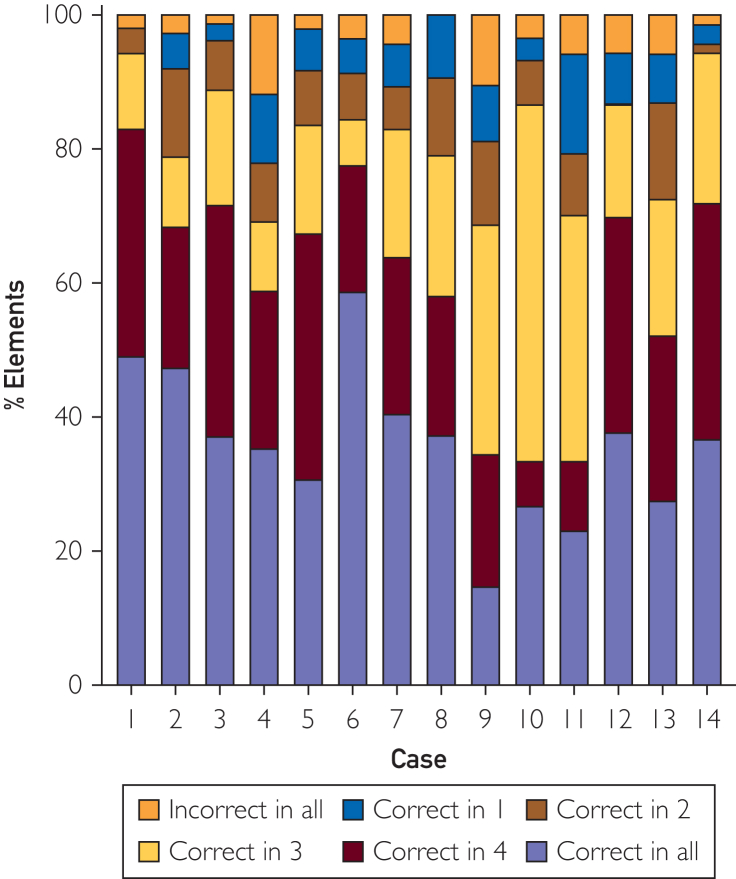
Percentage of clinical elements according to number of applications (0-5) in which the element was correctly captured for each case.

Regarding error severity, a mean of 3.0 (95% CI 0-4, range 0-21, Supplemental Table 3, [available online at https://www.mcpdigitalhealth.org/]) errors per case had potential to cause moderate-to-severe harm (AHRQ≥2). Platform E had a higher proportion of AHRQ≥2 errors than platform C (*P*=.0066). Weighted harm score for application E was significantly higher than applications A through D (*P*<.0082 for all comparisons), with no significant differences among the remaining applications (Table 1). Looking at cross-platform consistency for individual case elements, there was notable variation in elements captured across platforms, with a mean of only 35.8%±11.3% reported correctly by all 5 systems (Figure 4). Conversely, 4.3%±3.4% of elements were omitted or captured erroneously by all 5 systems, whereas the remaining 59.9%±10.2% were reported variably across platforms.

Qualitative analysis of clinical note errors revealed several recurring themes across platforms, including undersynthesis, misgendering, hallucination, medication errors, and substitution (Supplemental Table 4, [available online at https://www.mcpdigitalhealth.org/]). In several cases, ADS platforms failed to synthesize clinical history and examination findings into cohesive assessments, despite clinicians explicitly verbalizing their suspected diagnosis and reasoning. Misgendering of patients was also observed and appeared to be context dependent. However, most platforms allowed manual gender designation if the user wished to override the automated assignment. Although relatively infrequent, hallucinations resulted in the inclusion of fabricated test results and subjective commentary on a patient’s memory and medication adherence. Medication-related errors were the most common, with errors of both commission and omission observed across all platforms. Many of these appeared to be propagated from transcription errors, indicating deficiencies in terminology recognition at the ASR level. Platform E was particularly susceptible to substitution errors, in which a clinical term or phrase was replaced with an unrelated but contextually plausible alternative.

Qualitative analysis of the 13 observed errors with potential for severe harm or death (AHRQ>3) provided additional insight regarding clinical risk profile. Five (38%) of these severe errors were medication-related, 9 (69%) involved omissions, and nearly half (46%) were observed in notes generated by platform E. In the case involving a pregnant patient with decreased fetal movement (#12), both platforms B and E omitted a patient-reported aspirin allergy. In the simulated diabetic ketoacidosis case (#11), platform D omitted the physical examination observation of lethargy indicating altered mental status, while platform E stated that the patient was alert. In case 8 (incarcerated inguinal hernia), platform D omitted the patient-reported medication Stalevo and replaced entacapone with an anticonvulsant.

The only error designated as carrying risk of death (AHRQ=4) was observed in the case of pneumonia with sepsis (#13). Although the provider-articulated treatment plan included immediate hospital admission and initiation of 4.5 g of piperacillin-tazobactam, the note generated by platform E stated that admission would be considered and antibiotics would only be initiated if infection confirmed, with no mention of antibiotic agent or dose.

## Discussion

In this study, we observed significant variation in the accuracy, quality, and potential harm risk of clinical notes generated by 5 ADS platforms using simulated ambulatory encounters. These differences were observed across platforms and cases, indicating inter-platform and intra-platform variability. By analyzing transcript and clinical note output independently, we identified potential sources of error introduced at both the ASR and LLM levels.

We observed relevant variation in transcript accuracy, with a broad range in performance among platforms and cases. However, error transmission to the clinical note remained relatively consistent across platforms at ∼20%. This observation suggests that incorporating weights from LLM training may partially compensate for omissions and substitutions caused by speech recognition errors. Analysis of clinical note output revealed additional quality and safety issues at the LLM processing level. Approximately 25% of key clinical elements were omitted or captured erroneously, with wide variation among platforms and cases. Notably, we observed considerable inconsistency in errors across platforms, with only 35% of clinical elements correctly captured by all 5 platforms. This heterogeneity in model output is likely attributable in part to platform-specific differences in model training, fine tuning, and developer-specified templates. These findings report the unpredictability of ADS output, which limits the ability to make broadly applicable conclusions about the nature and type of errors that may be observed for any individual ADS platform.

Evaluation of error subtypes and qualitative categories raised additional considerations. Errors of omission were the most frequently observed subtype across the platforms, comprising approximately three-fourths of all errors. Omission errors may be particularly challenging to correct in clinical practice, as this necessitates both recognition and recall of missing encounter details. Furthermore, observation of medication errors across all platforms indicates an ecumenical need for targeted training on pharmaceutical terminology. Our observations of misgendering, errors in diagnostic synthesis, and critical transcription errors reflect those reported by clinician users in previous qualitative studies.^20^^,^^21^ These findings highlight potential areas of concern and emphasize the need for clinical expert involvement in ADS development.

Previous studies evaluating ADS performance have predominantly focused on postimplementation effects, with recent studies demonstrating beneficial impact on documentation efficiency and wellbeing.^4^, ^5^, ^6^, ^7^, ^8^^,^^22^, ^23^, ^24^ However, objective evaluation of ADS quality and safety has been relatively limited. In a simulation-based evaluation of 2 ADS platforms, physician graders identified an average of 2.88 errors per ADS-generated note, with errors of omission occurring most frequently across both platforms.^13^ In our previous work using ChatGPT to generate clinical notes using uploaded transcripts, we identified potential accuracy and consistency concerns, with only 53% of clinical elements reported correctly across 3 replicates.^16^ Several studies evaluating the safety of other medical LLM platforms have also yielded mixed findings. In a study evaluating handoff notes from emergency physicians to receiving inpatient teams, LLM-generated summaries received lower usefulness and patient safety ratings compared to physician-written notes, though the authors noted that none of the LLM errors constituted critical safety risks.^25^ Chen et al^12^ evaluated the harm risk of LLM responses to patient messages, observing a risk of severe harm in 7.1% of responses and death in 0.6%. These findings highlight the need for human oversight of medical documentation created using generative artificial intelligence technology.

Although physicians are ultimately responsible for the content of their clinical documentation, previous work utilizing other clinician-in-the-loop methods of documentation suggests that provider proofreading is both inconsistent and imperfect. Evaluation of clinical notes generated using ambient speech recognition found persistent, clinically significant errors after manual review by both a professional medical transcriptionist and physician.^26^ Errors have been observed in 4.8%-71% of signed notes generated using ambient dictation.^27^ Furthermore, previous work on automation bias suggests a tendency toward overreliance on LLM output, which may lead to poor compliance with manual review.^28^, ^29^, ^30^ In a cross-sectional simulation study evaluating provider editing of AI-generated draft responses to patient portal messages, participants frequently failed to recognize clinically relevant note errors.^15^ Because of ADS licensing costs, providers may experience explicit or implicit organizational pressure to increase their clinical output, which could further compound this problem.^31^ Our findings emphasize the necessity of manual review given the potential for clinically relevant errors observed across platforms.

The rapid market expansion of ADS technology with relatively little regulatory oversight underscores the urgent need for standardized, objective evaluation tools and governance models.^32^ In addition to these minimum universal standards, clinical practice groups must enact tailored governance policies around clinical use and human oversight requirements at the organizational level. Although some vendors use quality assessment tools such as PDQI-9 and DeepScore (DeepScribe),^33^ lack of standardization limits cross-platform evaluation. Effective evaluation must incorporate objective, reproducible metrics, including performance assessments for ASR and LLM output, error rate analysis, and provider perception of functional quality, readability, and utility. Postimplementation assessments should include regular quality and safety monitoring along with user feedback. Establishing a uniform, validated methodology for ADS evaluation is essential to ensure safety, accuracy, and reliability across platforms.

Our simulation approach facilitates objective, replicable evaluation of the quality, accuracy, and safety of both ADS-generated transcripts and clinical notes. We have previously demonstrated the utility of this method in assessing the quality and accuracy of notes generated by human scribes and ChatGPT4.^16^^,^^34^ Other authors have utilized similar simulation-based approaches for ADS performance testing, with inconsistent methods for evaluating the resultant output.^13^^,^^35^ In contrast to previous work, our rubric-based approach facilitates consistent, objective evaluation across various clinical scenarios.

This study has several limitations. We acknowledge that these 14 cases capture a limited representation of the potential variation in the ambulatory clinical setting. Understanding the risk profile of this technology will require an expanded testing protocol with a wider range of clinical settings, specialties, and challenging circumstances (e.g., >2 speakers, disagreement between participants, multilingual conversations, and ambient noise). In addition, our simulation testing was performed under optimized capture conditions utilizing prerecorded audio free of background noise. Hence, these results should not be directly extrapolated to real-world performance. Because of the lack of vendor transparency around details of platform development, including training strategies, model parameters, and fine tuning, we were unable to perform a more granular evaluation of potential model covariates. Finally, our study approximates the worst case scenario by evaluating the potential harm of unedited ADS-generated notes without reference to previous data. In the clinical setting, provider proofreading and reference to other data sources such as the electronic health record may reduce errors and mitigate potential harm. The effectiveness of these measures in the clinical setting is currently unknown and remains a target for future research efforts.

## Conclusion

Our findings provide essential insight into the potential for relevant variation in the quality, accuracy, and safety of output generated by ADS platforms. Although these tools have reported potential for improving documentation efficiency and physician wellbeing, their impact on patient safety remains unknown. With the growing number of commercially available ADS platforms, it is imperative to establish transparent assessment and reporting standards to empower informed adoption decisions. Our simulation-based evaluation methods provide an objective framework for comparative assessment of the performance and potential risks of ADS platforms.

## Potential Competing Interests

The authors report no competing interests.

## Ethics Statement

Institutional review board approval was not required for this study, as no human subjects or patient data were included.

## References

[1] Arndt B.G., Beasley J.W., Watkinson M.D. (2017). Tethered to the EHR: primary care physician workload assessment using EHR event log data and time-motion observations. Ann Fam Med.

[2] Gaffney A., Woolhandler S., Cai C. (2022). Medical documentation burden among US office-based physicians in 2019: A national study. JAMA Intern Med.

[3] Gardner R.L., Cooper E., Haskell J. (2019). Physician stress and burnout: the impact of health information technology. J Am Med Inform Assoc.

[4] Tierney A.A., Gayre G., Hoberman B. (2024). Ambient artificial intelligence scribes to alleviate the burden of clinical documentation. NEJM Catalyst.

[5] Ma S.P., Liang A.S., Shah S.J. (2025). Ambient artificial intelligence scribes: utilization and impact on documentation time. J Am Med Inform Assoc.

[6] Misurac J., Knake L.A., Blum J.M. (2025). The effect of ambient artificial intelligence notes on provider burnout. Appl Clin Inform.

[7] Balloch J., Sridharan S., Oldham G. (2024). Use of an ambient artificial intelligence tool to improve quality of clinical documentation. Future Healthc J.

[8] Shah S.J., Devon-Sand A., Ma S.P. (2025). Ambient artificial intelligence scribes: physician burnout and perspectives on usability and documentation burden. J Am Med Inform Assoc.

[9] Ambient technology’s role in the ongoing AI revolution in healthcare. Med. Group Manag. Assoc. https://www.mgma.com/mgma-stat/ambient-technologys-role-in-the-ai-revolution.

[10] AI Ambient Scribes. Elion. https://elion.health/categories/ai-ambient-scribes/products.

[11] Topaz M., Schaffer A., Lai K.H., Korach Z.T., Einbinder J., Zhou L. (2018). Medical malpractice trends: errors in automated speech recognition. J Med Syst.

[12] Chen S., Guevara M., Moningi S. (2024). The effect of using a large language model to respond to patient messages. Lancet Digit Health.

[13] Biro J.M., Handley J.L., Mickler J. (2025). The value of simulation testing for the evaluation of ambient digital scribes: a case report. J Am Med Inform Assoc.

[14] Lin Z., Guan S., Zhang W., Zhang H., Li Y., Zhang H. (2024). Towards trustworthy LLMs: a review on debiasing and dehallucinating in large language models. Artif Intell Rev.

[15] Biro J.M., Handley J.L., Malcolm McCurry J. (2025). Opportunities and risks of artificial intelligence in patient portal messaging in primary care. NPJ Digit Med.

[16] Kernberg A., Gold J.A., Mohan V. (2024). Using ChatGPT-4 to create structured medical notes from audio recordings of physician-patient encounters: comparative study. J Med Internet Res.

[17] Stetson P.D., Bakken S., Wrenn J.O., Siegler E.L. (2012). Assessing electronic note quality using the physician documentation quality instrument (PDQI-9). Appl Clin Inform.

[18] Hose B.Z., Handley J.L., Biro J. (2025). Development of a preliminary patient safety classification system for generative AI. BMJ Qual Saf.

[19] Agency for Healthcare Research and Quality (2018). Common formats for event reporting - hospital version 2.0a. https://www.psoppc.org/psoppc_web/publicpages/commonFormatsHV2.0.

[20] Bundy H., Gerhart J., Baek S. (2024). Can the administrative loads of physicians be alleviated by AI-facilitated clinical documentation?. J Gen Intern Med.

[21] Cain C.H., Davis A.C., Broder B. (2025). Quality assurance during the rapid implementation of an AI-assisted clinical documentation support tool. NEJM AI.

[22] Duggan M.J., Gervase J., Schoenbaum A. (2025). Clinician experiences with ambient scribe technology to assist with documentation burden and efficiency. JAMA Netw Open.

[23] Liu T.-L., Hetherington T.C., Dharod A. (2024). Does AI-powered clinical documentation enhance clinician efficiency? a longitudinal study. NEJM AI.

[24] Albrecht M., Shanks D., Shah T. (2025). Enhancing clinical documentation with ambient artificial intelligence: a quality improvement survey assessing clinician perspectives on work burden, burnout, and job satisfaction. JAMIA Open.

[25] Hartman V., Zhang X., Poddar R. (2024). Developing and evaluating large language model–generated emergency medicine handoff notes. JAMA Netw Open.

[26] Zhou L., Blackley S.V., Kowalski L. (2018). Analysis of errors in dictated clinical documents assisted by speech recognition software and professional transcriptionists. JAMA Netw Open.

[27] Blackley S.V., Huynh J., Wang L., Korach Z., Zhou L. (2019). Speech recognition for clinical documentation from 1990 to 2018: a systematic review. J Am Med Inf Assoc.

[28] Tucci V., Saary J., Doyle T.E. (2022). Factors influencing trust in medical artificial intelligence for healthcare professionals: a narrative review. J Med Artif Intell.

[29] Duran J.M., Jongsma K.R. (2021). Who is afraid of black box algorithms? On the epistemological and ethical basis of trust in medical AI. J Med Ethics.

[30] Wang D.Y., Ding J., Sun A.L. (2023). Artificial intelligence suppression as a strategy to mitigate artificial intelligence automation bias. J Am Med Inform Assoc.

[31] Burden M., Dyrbye L. (2025). Evidence-based work design—bridging the divide. N Engl J Med.

[32] Wang H., Yang R., Alwakeel M. (2025). An evaluation framework for ambient digital scribing tools in clinical applications. NPJ Digit Med.

[33] Oleson J. (2024).

[34] Pranaat R., Mohan V., O’Reilly M. (2017). Use of simulation based on an electronic health records environment to evaluate the structure and accuracy of notes generated by medical scribes: proof-of-concept study. JMIR Med Inform.

[35] van Buchem M.M., Kant I.M.J., King L., Kazmaier J., Steyerberg E.W., Bauer M.P. (2024). Impact of a Digital Scribe System on Clinical Documentation Time and Quality: Usability Study. JMIR AI.

