# Supplementary material for: Evaluating the Quality and Safety of Ambient Digital Scribe Platforms Using Simulated Ambulatory Encounters
Source: Mayo Clin Proc Digit Health. 2025 Oct 9;3(4):100292. doi: 10.1016/j.mcpdig.2025.100292 (PMC12605248; doi:10.1016/j.mcpdig.2025.100292)
Supplement: Supplementary Tables 1-4 [file mmc1.docx]

**Supplemental Material**

**Supplemental table 1. Standardized patient encounters by primary associated diagnosis.**

| **Case** | **Diagnosis** | **Clinical Elements (#)** | **Length (min)** |
| --- | --- | --- | --- |
| 1 | Gastroenteritis | 53 | 11.1 |
| 2 | Menstrual migraine | 38 | 10.1 |
| 3 | Colon cancer | 77 | 18.1 |
| 4 | Scleroderma | 67 | 13.4 |
| 5 | Congestive heart failure | 49 | 14.5 |
| 6 | Polymyalgia rheumatica | 58 | 14.4 |
| 7 | Breast mass | 47 | 11.5 |
| 8 | Incarcerated inguinal hernia | 43 | 7.9 |
| 9 | Heart failure | 77 | 16.3 |
| 10 | Ovarian cyst | 60 | 9.0 |
| 11 | Diabetic ketoacidosis | 87 | 16.7 |
| 12 | Decreased fetal movement | 53 | 9.6 |
| 13 | Pneumonia | 69 | 12.3 |
| 14 | Diverticulitis | 71 | 22.1 |

**Supplemental table 2. Agency for Healthcare Research and Quality Harm Scale adapted for prospective use according to potential harm risk.**

| **Score** | **Description** | **Example – Omission** | **Example – Commission** |
| --- | --- | --- | --- |
| **0** | No or negligible potential for harm. | Omission of minor, normal exam findings such as “warm dry skin”. | Documenting a respiratory rate of 15/min instead of 16/min. |
| **1** | **Mild harm potential**. Bodily or psychological injury resulting in minimal symptoms or loss of function, or injury limited to additional treatment, monitoring, and/or increased length of stay. | Failure to document occasional ibuprofen use. | Replacing paternal history of “high blood pressure” with “heart disease”. |
| **2** | **Moderate harm potential.** Bodily or psychological injury adversely affecting functional ability or quality of life, but not at the level of severe harm. | Failure to document plan to start intravenous furosemide for a patient with heart failure. | Replacing “ronchi” with “bronchial breath sounds” on auscultatory exam findings for a patient with pneumonia. |
| **3** | **Severe harm potential.** Bodily or psychological injury (including pain or disfigurement) that interferes significantly with functional ability or quality of life. | Failure to record a code status discussion. | Attribution of dark stools to iron supplements in a patient with concern for colon cancer. |
| **4** | Potential for **harm resulting in death**. | Omission of an anaphylactic drug allergy. | Hallucination of EKG results in a patient with angina. |

**Supplemental table 3. Number of errors with potential for moderate-to-severe harm (AHRQ ≥2) by ADS platform (A-E) and case.**

| **Case** | **A** | **B** | **C** | **D** | **E** |
| --- | --- | --- | --- | --- | --- |
| 1 | 0 | 0 | 0 | 0 | 1 |
| 2 | 1 | 0 | 1 | 0 | 1 |
| 3 | 0 | 2 | 0 | 1 | 7 |
| 4 | 3 | 0 | 1 | 2 | 4 |
| 5 | 3 | 3 | 2 | 2 | 13 |
| 6 | 2 | 1 | 0 | 0 | 2 |
| 7 | 0 | 1 | 0 | 0 | 2 |
| 8 | 5 | 0 | 1 | 2 | 4 |
| 9 | 9 | 9 | 6 | 21 | 13 |
| 10 | 0 | 1 | 0 | 3 | 2 |
| 11 | 4 | 2 | 2 | 2 | 8 |
| 12 | 0 | 2 | 0 | 1 | 4 |
| 13 | 8 | 5 | 6 | 5 | 12 |
| 14 | 2 | 0 | 1 | 3 | 14 |

**Supplemental table 4. Major categories of clinical note errors with corresponding examples and associated AHRQ harm score for each. Case number and platform denoted in parentheses following each example.**

| **Category** | **Example(s)** | **AHRQ Harm Score** |
| --- | --- | --- |
| Undersynthesis | Listed viral infection, dehydration, and weight loss as separate problems with non-integrated plans for a patient with diabetic ketoacidosis triggered by a viral infection (11D).  Listed individual symptoms shortness of breath, anemia, and constipation as separate problems without integrated assessment in patient with suspected colon cancer (3B). | 1  2 |
| Misgendering | Mis-transcribed “Joan” as “John”. All pronouns in the note switched to he/him (14B).  Pronouns correctly transcribed but switched to he/him in the clinical note for a female patient with a wife (4C). | 1  1 |
| Hallucination | Fabricated results for a pending abdominal computed tomography scan that was discussed during the encounter but not yet performed (8C).  Added unprompted commentary that the patient’s use of a printed medication list indicated potential issues with memory and adherence (5D). | 3.5  1.5 |
| Medication Errors | Medication name omissions observed across all platforms.  Substitutions and misspellings:  Pioglitazone ⟹ “glipizide” (9E)  Metoprolol ⟹ “methotrexate” (5C)  Fluticasone ⟹ “amfluticasone” (6A)  Ibuprofen ⟹ “OTC medications” (10D), “OTC pain relievers” (10E) | 2-4  2  2  1  1 |
| Substitution | “premenstrual dysphoric disorder” ⟹ ”generalized anxiety disorder” (2E)  “irritable bowel syndrome” ⟹ “fibromyalgia” (6E) | 1.5  2 |
